# Effect of Cadherin-11 on the Proliferation, Migration, and ECM Synthesis of Chondrocyte

**DOI:** 10.1155/2023/9985334

**Published:** 2023-05-26

**Authors:** Jia Li, Hang Shi, Xia Liu, Haiyue Jiang

**Affiliations:** Plastic Surgery Hospital, Chinese Academy of Medical Sciences & Peking Union Medical College, 33 Ba-da-chu Road, Shijingshan District, Beijing 100144, China

## Abstract

Nonsyndromic microtia is a kind of congenital ear malformation with unclear pathogenic genes. Cadherin-11 (CDH11, OB-cadherin) is a member of the cadherin family, which has been demonstrated to play important roles in controlling morphogenesis and cell biological characteristics during multiple developmental processes. In the present study, we found low expression of CDH11 in microtia cartilage compared with the normal one for the first time. For a more comprehensive and in-depth understanding of CDH11 in microtia development, we performed both gain- and loss-of-function experiments to detect the effect of CDH11 on chondrocytes. CDH11 promoted chondrocyte proliferation by increasing S-phase cell numbers and increasing cell migration, which is important for tissue morphogenesis. Additionally, knockdown of CDH11 in chondrocytes reduced the quality of engineered cartilage by decreasing the key transcription factors of chondrogenesis, SOX9 expression, and cartilage ECM production, including collagen type II (COL2A) and elastin (ELN), compared to the control group. Furthermore, RNA-Seq on CDH11 knockdown chondrocytes showed that it was highly related to HOX family genes, which have been reported to be important regulatory genes patterning craniofacial tissue formation. This study identified CDH11 as a candidate pathogenic gene of microtia and supported the potential key role of CDH11 in craniofacial malformations.

## 1. Introduction

Microtia is a congenital malformation of the external and middle ear caused by the abnormal development of the first and second visceral arch and the first sulcus [1, 2]. Although it is believed that both genetic and environmental factors may play an important role in microtia, there is currently no consensus concerning the pathogenesis and etiology of microtia [3, 4]. It is important to identify key pathogenic genes for early diagnosis as well as new therapeutic targets.

Cadherins are transmembrane Ca^2+^-dependent homophilic cell adhesion receptors, which are vital for the proliferation, differentiation, migration, and maintenance of structural integrity of tissues and homeostasis [5, 6]. They also play important roles in the recognition and sorting of cells during development [7]. The members of the cadherin are emerging as susceptibility genes in multifactorial disorders and cancers [8, 9]. Among them, CDH11, a type II cadherin, is widely expressed in mesenchymal cell types including, smooth muscle cells (SMCs), fibroblasts, and osteoblasts [10]. CDH11 plays important roles in tumor and metabolic and inflammatory diseases [11, 12]. In addition, heterozygous variants in CDH11, which decrease cell-cell adhesion and increase cell migratory behavior, cause a form of Teebi hypertelorism syndrome (THS), a rare craniofacial disorder characterized by hypertelorism, prominent forehead, and short nose with broad or depressed nasal root [13]. Until now, four distinct cadherins have been implicated in human Mendelian disorders, mainly featuring skin, retinal, and hearing manifestations, and a novel loss-of-function variant in CDH11 has been identified as a cause of BSGS (brainstem gliomas, BSGs). These results support the role of cadherin-11 as a key player in axial and craniofacial malformations [14].

Previous studies from our group revealed that CDH11 played a promoting role in the production of proteoglycans, COL2A, and ELN in cartilage and functioned as an important regulator of the microstructure of cartilage ECM. In the present study, the data demonstrated that CDH11 promoted chondrocyte proliferation, migration, and tissue formation in microtia.

## 2. Materials and Methods

### 2.1. Animal and Tissue Collection

All procedures were approved by the Research Ethics Committee at the Plastic Surgery Hospital of the Chinese Academy of Medical Sciences and Peking Union Medical College (Beijing, China). Human microtia cartilage tissue was harvested from remnant auricular cartilage of microtia patients (*n* = 4, between 5 and 8 years) undergoing the first stage of ear reconstructive surgery, and normal auricle cartilage tissues (*n* = 2, between 15 and 28 years) were from the patients undergoing rhinoplasty with autologous ear cartilage in our hospital. Pig ears cartilages were taken from miniature pigs (*n* = 9, between 5 and 7 months).

### 2.2. Chondrocyte Isolation and Culture

The harvested porcine ear cartilage was carefully dissected to remove fibrous tissue and perichondrium, then fragmented into 1 mm^3^ pieces, washed in phosphate-buffered saline (PBS) solution containing 100 U/mL penicillin and 100 *μ*g/mL streptomycin, added by 0.25% pancreatin, shaken at 37°C and 80 rpm for 30 min, discarded the supernatant, and treated with 0.2% type IV collagenase (Sigma -Aldrich, St. Louis, MO, USA), shaken at 37°C, 80 rpm for 8 h. The isolated cells were cultured and expanded in a complete medium (DMEM, Gibco, Grand Island, NY, USA) containing 10% fetal bovine serum (FBS, Gibco, USA) and 1% penicillin (Corning, USA) at 37°C with 95% humidity and 5% CO_2_.

### 2.3. Transcriptome Sequencing Analysis

Total RNA was extracted from the human chondrocytes using the TRIzol reagent (Life Technologies, Carlsbad, CA, USA), and the integrity was assessed using the RNA Nano 6000 Assay Kit of the Bioanalyzer 2100 system (Agilent Technologies, CA, USA). RNA-seq library preparation and sequencing were performed at the Novogene Bioinformatics Technology Co., Ltd. (Beijing, China).

#### 2.3.1. Data Analysis

The UMI (unique molecular identifiers) was extracted by UMI-tools v1.0.0. The downstream analyses were based on clean UMI reads with high quality. HTSeq v0.9.1 was used to count the read numbers mapped to each gene. Differential expression analysis of two conditions/groups (two biological replicates per condition) was performed using the DESeq R package (1.18.0).

The differential expression genes were determined by an adjusted P value <0.05. GO terms with a corrected-value <0.05 were considered significantly enriched by differentially expressed genes. Clusters of orthologous groups and pathway analyses were performed using the KEGG (http://www.genome.jp/kegg) analytical tool. We used KOBAS software to test the statistical enrichment of differential expression genes in KEGG pathways.

### 2.4. Tissue-Engineered Cartilage Construction

The porcine chondrocytes transfected with shCDH11 and scramble were resuspended in 30% Pluronic F-127 at an ultimate concentration of 5 × 10^7^ cells/ml and injected subcutaneously (0.5 ml/dot, *n* = 5) into BALB/c-nude mice as the shCDH11 group and the shScramble group, respectively. The engineered cartilage grafts were harvested after 4 w in vivo for the follow-up assay.

### 2.5. Histological Examination of Cartilage Tissue

The human ear cartilage and porcine tissue-engineered cartilage were fixed in 4% paraformaldehyde, dehydrated in a gradient ethanol series, and then embedded in paraffin. Specimens were stained with hematoxylin-eosin staining (HE) and special staining, including Masson tricolor staining (G1006, Servicebio), Elastica Van Gieson (EVG) staining (G1042, Servicebio), Alcian blue (G1027, Servicebio) staining, and Safranin O staining (G1053, Servicebio). For immunohistochemical examination, the primary antibody against CDH11 (Ab151302, Abcom) and the second antibody (GB23303, Servicebio) were used according to the technical manual, followed by color development, and the pictures were taken under a light microscope (CX23, OLYMPUS, Japan).

### 2.6. Immunofluorescence Assay

The tissue slices were dewaxed in gradient alcohol and placed in a citric acid (G1202, Servicebio, PH6.0) solution for antigen retrieval. For immunofluorescent staining, the primary antibody against CDH11 (Ab151302, Abcom), COL2A (Ab34712, Abcam), ELN (Ab21610, Abcam), and SOX9 (Ab185966, Abcam), and the secondary antibody (GB21303, Servicebio) were used. Cell nuclei were counterstained with DAPI (G1012, Servicebio) for 8 min at room temperature in the dark. The sections were sealed with antifluorescence quenching (G1401, Servicebio) sealing tablets, and the images were acquired using an ultrahigh-resolution microscope (Nikon Eclipse C1, Japan).

### 2.7. Lentivirus Vector-Mediated CDH11 Knockdown and Overexpression

According to the target gene sequence, the CDH11 overexpression and shRNA plasmid were designed and obtained from Syngentech Co., Ltd (Beijing, China). HEK 293T cells were used for lentiviral packaging transfection. The transfection efficiency was detected by quantitative polymerase chain reaction (qRT-PCR) after 72 h.

### 2.8. Chondrocytes Proliferation Assay

The chondrocytes were cultured in a 96-well plate with a density of 2 × 10^3^ cells/well for 7 days. Each well was incubated with 10 *μ*L CCK-8 (P04D19, GPL, China) solution for 2 h at 37°C with 5% CO_2_. The absorbance was then measured at 450 nm by a microplate reader (Thermo Labsystems, Vantaa, Finland), and GraphPad Prism 8.0 software was used to draw the proliferation curve.

### 2.9. Chondrocyte Cell Cycle Assay

The cell cycle status was assessed by flow cytometry using a Cell Cycle and Apoptosis Analysis kit (containing RNase A, P04D16, China). The cells were digested with trypsin and fixed by 95% ethanol at 4°C for 12 h. After being rinsed with PBS, the cells were stained with propidium iodide (PI) buffer (containing 0.4 ml staining buffer, 15 *μ*L of 25X PI, and 4 *μ*L of 2.5 mg/ml RNase A) for 30 min. Flow cytometry (ACEA, NovoCyte, 3130) was used to assess the cell cycle distribution.

### 2.10. Chondrocytes Migration Assay

For the wound healing test, the chondrocytes were cultured in a 6-well (5 × 10^5^ cells/well) until reaching 90% confluence. A horizontal scratch was made on the cell monolayer using a 200 *μ*L pipette tip. Wound healing was observed at 0 h, 24 h, and 48 h. Images of the cells were captured using an inverted microscope (TE2000-S, Nikon), and the area percentage of cell migration was measured using the Image J software. The percentage of the cell migration rate was quantified as (scratch area 0 h − scratch area *x *h)/scratch area 0 h ∗ 100%.

For the transwell test, cell suspensions were prepared in a serum-free medium at a final concentration of 3 × 10^5^ cells/ml and added to the upper chambers. 500 *μ*L culture medium containing 20% FBS was added in the lower chamber. After being incubated at 37°C for 24 hours, the cells were fixed with 4% paraformaldehyde for 20 min, scratched on the top membrane by cotton, and stained with 1% crystal violet for 10 min. The pictures were taken under an inverted microscope (TI2-U, Nikon), and the membrane of the transwell chamber was soaked in 200 *μ*L glacial acetic acid to decolorize, and detected with a microplate reader (Thermo Labsystems, Vantaa, Finland) at 570 nm.

### 2.11. Quantitative Real-Time Polymerase Chain Reaction (qRT-PCR)

Total RNA was extracted using a Trizol reagent (Life Technologies), and RNA was reverse transcribed to cDNA using a reverse transcription kit (Invitrogen Corp., Carlsbad, CA, USA). qRT-PCR analysis was performed with a Fast SYBR Green I Real-Time PCR system (Bio-Rad, Hercules, CA, USA) according to the manufacturer's instructions with the gene expression of CDH11, SOX9, COL2A, and ELN. Primer sequences are summarized in Table 1. The relative gene expression levels were calculated by the 2^−ΔΔ*Ct*^ method.

### 2.12. Western Blot Analysis

The engineered cartilage grafts harvested after 4w in vivo were washed twice with PBS and lysed with RIPA lysis reagent (G2002, Servicebio). The protein concentration was determined with a BCA protein assay kit (G2026, Servicebio). Extracts were resolved by SDS-PAGE, electroblotted onto nitrocellulose membranes, and incubated with primary antibodies against CDH11 (32–1700, 85KD, Thermo Fisher, MA, USA), SOX9 (Ab185966, 70KD, Abcam), COL2A (Ab34712, 117KD, Abcam), ELN (Ab9519, 70KD, Abcam), and *β*-Actin (GB12001, 36KD, Servicebio) overnight at 4°C. Next, the membranes were incubated with horseradish peroxidase-conjugated secondary antibodies. Images were acquired and quantified with Alpha Ease FC software. *β*-Actin was used to normalize target proteins.

### 2.13. Statistical Analysis

The data are presented as the mean ± SD. The two-tailed Student's *t*-test was used to analyze the differences between the groups using GraphPad Prism 8.0 software. A value of *p* < 0.05 was statistically significant.

## 3. Results

### 3.1. Low Expression of CDH11 in Microtia Cartilage Compared with Normal Cartilage

**Figure 1:**
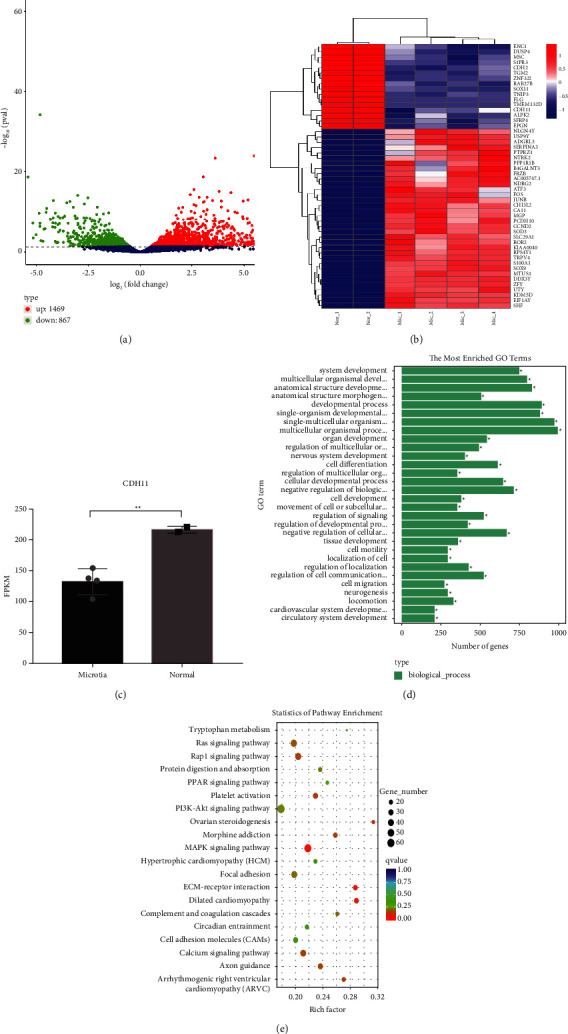
Low expression of CDH11 in microtia cartilage compared with normal cartilage. (a) Volcano map of differentially expressed genes. (b) Heat map of the microarray between microtia and normal cartilage. (c) The expression of CDH11 was measured by FPKM between microtia and normal. (d) Gene ontology (GO) analysis of DEGs. (e) KEGG pathway analysis of DEGs. (4 microtia patients, 2 normal human were used in this experiment).

To better understand the gene differential expression in microtia, transcriptome sequencing (RNA-Seq) analysis was performed between human microtia and normal auricle chondrocytes. Compared with the normal group, 2336 differentially expressed genes (DEGs) were detected, including 1469 upregulated and 867 downregulated genes (Figure 1(a)). Clearly, CDH11 was in these DEGs and from the fragments per kilobase million (FPKM) results. The expression of CDH11 was significantly downregulated in microtia compared with normal chondrocytes (*p* = 0.0062) (Figures 1(b) and 1(c)).

Gene ontology (GO) and Kyoto Encyclopedia of Genes and Genomes (KEGG) pathway analyses of the differentially expressed mRNAs were performed to determine the functions of the DEGs. A GO analysis showed that significantly enriched GO terms were mainly distributed in the biological processes (BP) categories, including multicellular organismal processes, single-multicellular organism processes, and developmental processes as the top three (Figure 1(d)). According to KEGG pathway annotation, the 20 most significantly enriched pathways were examined in detail, and the different genes were mostly enriched in ECM-receptor interaction, MAPK signaling pathway, PI3K-Akt signaling pathway, and so on (Figure 1(e)).

### 3.2. CDH11 is Expressed in Human Auricular Cartilage Tissue

**Figure 2:**
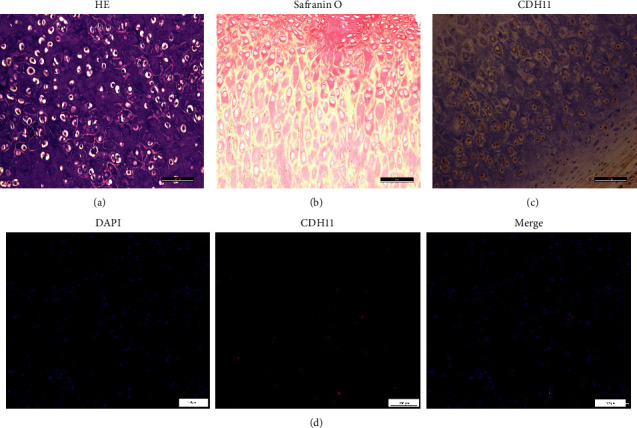
CDH11 is expressed in human microtia cartilage tissues and chondrocytes. (a) HE staining of sections of human microtia cartilage tissues (the scale bar indicates 100 *µ*m). (b) Safranin O staining of sections of human microtia cartilage tissues (the scale bar indicates 50 *µ*m). (c) Immunostaining of CDH11 in microtia cartilage tissue (the scale bar indicates 100 *µ*m). (d) The images of immunofluorescence staining for CDH11 in microtia chondrocytes (the scale bar indicates 50 *µ*m).

As illustrated in HE staining, human microtia auricular cartilage exhibited typical cartilage lacuna, which was disorderly arranged and surrounded by dense extracellular matrix (Figure 2(a)). Safranin O staining showed an overview of the macromolecules proteoglycan deposition in the tissue (Figure 2(b)). By immunohistochemistry staining, we found the positive expression of CDH11 in the microtia cartilage tissue (Figure 2(c)). The immunofluorescence staining of the microtia chondrocytes confirmed that CDH11 was expressed in the chondrocyte membrane (Figure 2(d)).

### 3.3. CDH11 Promotes the Proliferation of Chondrocytes by Increasing the S-Phase of the Chondrocyte Cell Cycle Assay In Vitro

**Figure 3:**
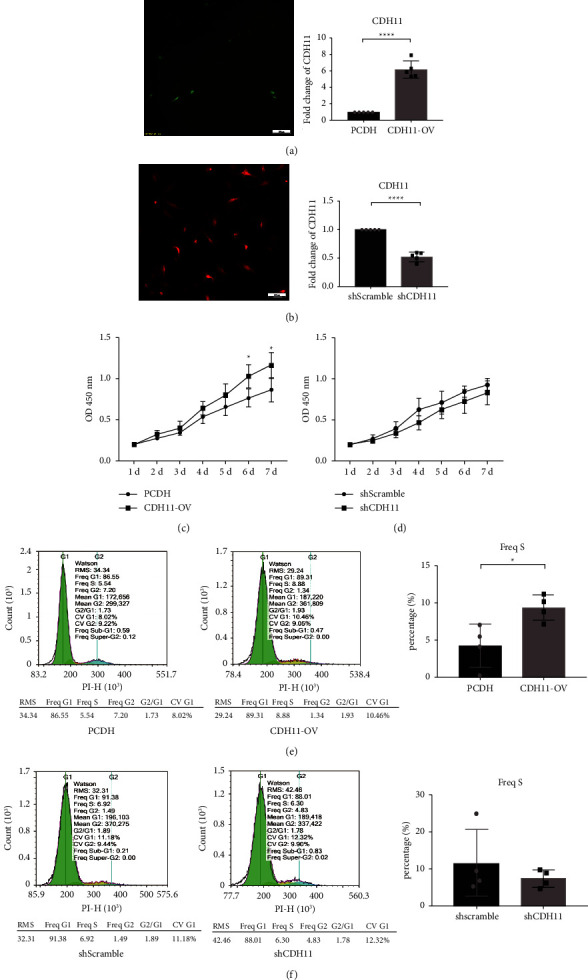
CDH11 promotes the proliferation of chondrocytes by increasing the S-phase of cell cycle in vitro. (a) CDH11 mRNA expression levels after overexpression plasmid transfection for 72 hours measured by qRT-PCR. (b) CDH11 mRNA expression levels after shRNA transfection for 72 hours measured by qRT-PCR. (c) The proliferation on the chondrocytes after the CDH11 overexpression was measured by CCK-8. (d) The proliferation on the chondrocytes after CDH11 knockdown was measured by CCK-8. (e) The percentage of each phase in cell cycle after the CDH11 overexpression was assessed by flow cytometry. (f) The percentage of each phase in cell cycle after CDH11 knockdown was assessed by flow cytometry. Porcine chondrocytes were used in this experiment. All experiments were performed at least three times. ^*∗*^*p* < 0.05; ^*∗∗∗∗*^*p* < 0.0001.

To explore the biological functions of CDH11 in chondrocytes, both gain- and loss-of-function experiments were performed in vitro. We employed an overexpression plasmid to stimulate high expression of CDH11 and used shRNA-mediated knockdown of CDH11 to study the effect of CDH11 on chondrocyte proliferation. After transfection for 72 h, CDH11 mRNA expression was about 6-fold higher in the CDH11-OV group (the CDH11 overexpression group) than that in the PCDH group (the control group), while that in the CDH11 knockdown group was 50% lower than that in the shScramble group (Figures 3(a) and 3(b)). Green fluorescence represents overexpressed transfected chondrocytes, and red fluorescence represents knockdown transfected chondrocytes.

The effect of CDH11 on chondrocyte proliferation was examined using CCK-8 assay. The present results showed that the CDH11 overexpression significantly promoted the proliferation of chondrocytes on 6 days and 7 days (*p* = 0.0104, 0.0123) (Figure 3(c)), while there was no significant difference between the shCDH11 group and the shScramble group (Figure 3(d)).

Furthermore, the chondrocyte cycle test by flow cytometry showed a significant increase in the S-phase chondrocyte number percentage in the CDH11-OV group compared with the PCDH group (*p* = 0.0232) (Figures 3(e) and 3(f)).

### 3.4. CDH11 Promotes the Migration of Chondrocytes In Vitro

**Figure 4:**
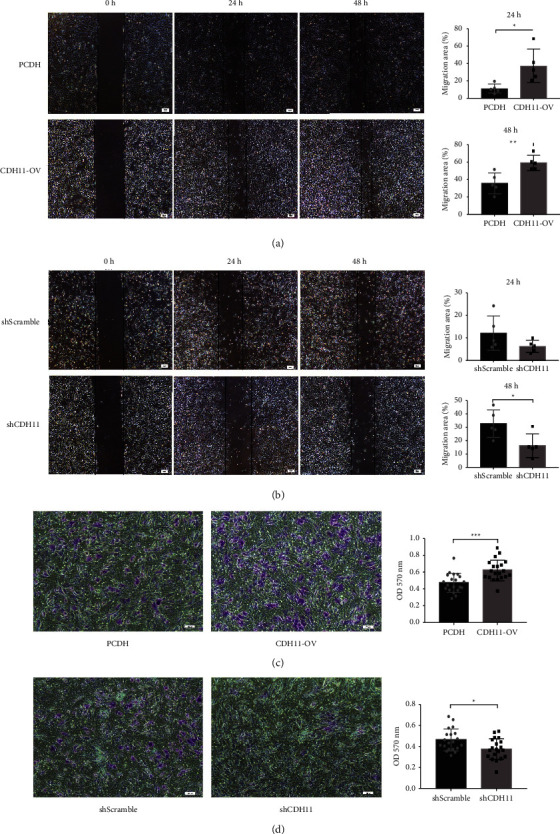
CDH11 promotes migration and invasion of chondrocytes in vitro. (a) The ability of migration was measured by wound healing after CDH11 overexpression. (b) The ability of migration was measured by wound healing after CDH11 knockdown. (c) The ability of invasion was measured by transwell after CDH11 overexpression. (d) The ability of invasion was measured by transwell after CDH11 knockdown. Porcine chondrocytes were used in this experiment. All experiments were performed at least three times. ^*∗*^*p* < 0.05; ^*∗∗*^*p* < 0.01.^*∗∗∗*^*p* < 0.001.

Chondrocyte migration is an important factor in tissue morphogenesis. To investigate the effects of CDH11 on the migration properties of chondrocytes, wound healing experiments were performed in vitro. The results showed that chondrocytes transfected with the CDH11 overexpression plasmid migrated faster than the control group in the CDH11-OV group at 24 h (Figure 4(a), *p* = 0.0170) and 48 h (Figure 4(a), *p* = 0.0073). CDH11 knockdown decreased the migration ability of chondrocytes in comparison to the control group at 48 h (Figure 4(b), *p* = 0.0274).

A transwell assay was performed to further study the effects of CDH11 on chondrocyte migration. CDH11 overexpression increased the invasive chondrocyte number (*p* = 0.0112), while CDH11 knockdown decreased the number of transmembrane chondrocytes at 24 h (Figures 4(c) and 4(d), *p* = 0.0230).

### 3.5. Knockdown of CDH11 Suppresses Engineered Cartilage Formation In Vivo

**Figure 5:**
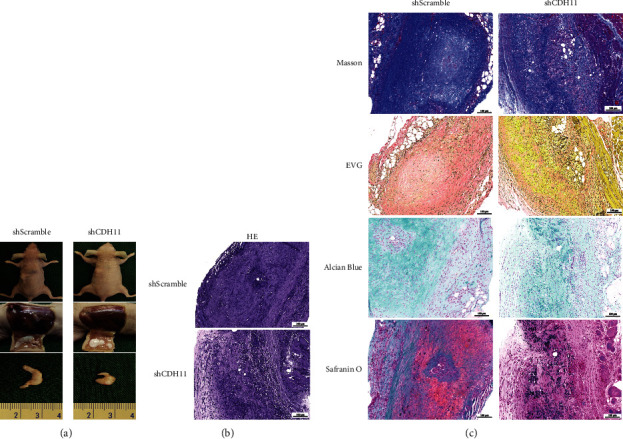
Knockdown of CDH11 suppresses engineered cartilage formation in vivo. (a) Macroscopic images of the engineered cartilage subcutaneously implanted for 4 w. (b) Hematoxylin and eosin staining for engineered cartilage formation (the scale bar indicates 100 *µ*m). (c) The Masson, EVG, safranin O, and alcian blue staining of the engineered cartilage (the scale bar indicates 100 *µ*m). Porcine chondrocytes were used in this experiment.

To further identify if CDH11 affects tissue formation in vivo, we used CDH11 knockdown chondrocytes and Pluronic F-127 to construct engineered cartilage in nude mice subcutaneously. After 4w in vivo, the volume and thickness of tissue-engineered cartilage formed in the shCDH11 group were grossly smaller and thinner than those in the shScramble group (Figure 5(a)). Histologically, HE staining results showed that the implants of the two groups developed into mature cartilage tissue with typical lacuna structures. However, the cartilage-like tissues in the shCDH11 group were fewer and looser than those in the control group, indicating that low CDH11 expression was less conducive to chondrocyte adhesion and tissue integrity (Figure 5(b)). Special staining was used to evaluate the synthesis of ECM. Masson's trichrome and EVG staining showed less expression of collagen and elastic fibers in the shCDH11 group than that in the shScramble group. Safranin O and Alcian blue staining results showed that there was lighter and more uneven distribution staining in the shCDH11 group than the control group, indicating that CDH11 knockdown leads to insufficient macromolecules proteoglycan formation (Figure 5(c)).

### 3.6. Knockdown of CDH11 Suppresses ECM Synthesis In Vivo

**Figure 6:**
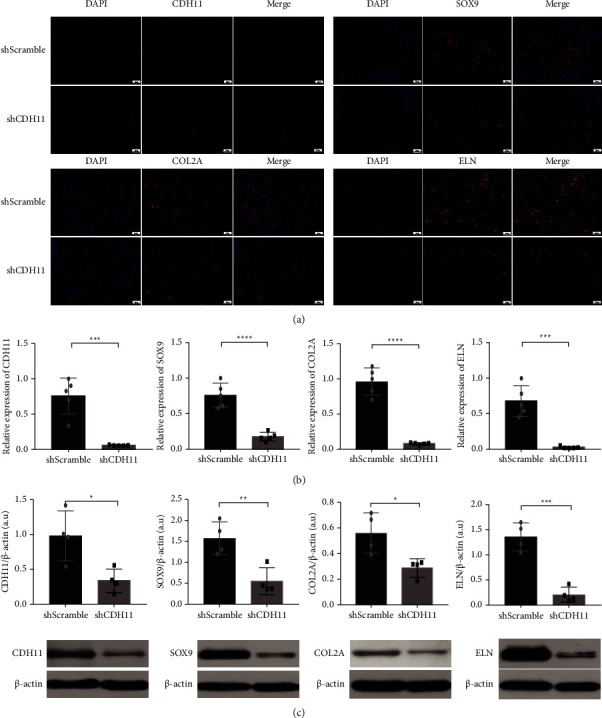
Knockdown of CDH11 suppresses ECM synthesis in vivo. (a) The images of CDH11, SOX9, COL2A, and ELN immunofluorescence in the shScramble and shCDH11 groups (the scale bar indicates 50 *µ*m). (b) Relative mRNA levels of CDH11, SOX9, COL2A, and ELN were measured by qPCR. (c) The protein levels of CDH11, SOX9, COL2A, and ELN were measured by western blot. Porcine chondrocytes were used in this experiment. All experiments were performed at least three times. ^*∗*^*p* < 0.05; ^*∗∗*^*p* < 0.01; ^*∗∗∗*^*p* < 0.001; ^*∗∗∗∗*^*p* < 0.0001.

The protein expression of COL2A and ELN, the main ECM components in cartilage tissue, was detected by immunofluorescence. The expression level of these proteins decreased in the shCDH11 group compared to the control group. Compared with the shScramble group, the expression of SOX9, the key transcription factor in chondrogenesis, was reduced (Figure 6(a)). Furthermore, we used qRT-PCR and western blot analysis to confirm the inhibition effects of CDH11 knockdown on chondrogenesis and ECM production at both mRNA and protein levels (Figures 6(b) and 6(c)).

### 3.7. CDH11 is Highly Correlated with HOX Family Genes

**Figure 7:**
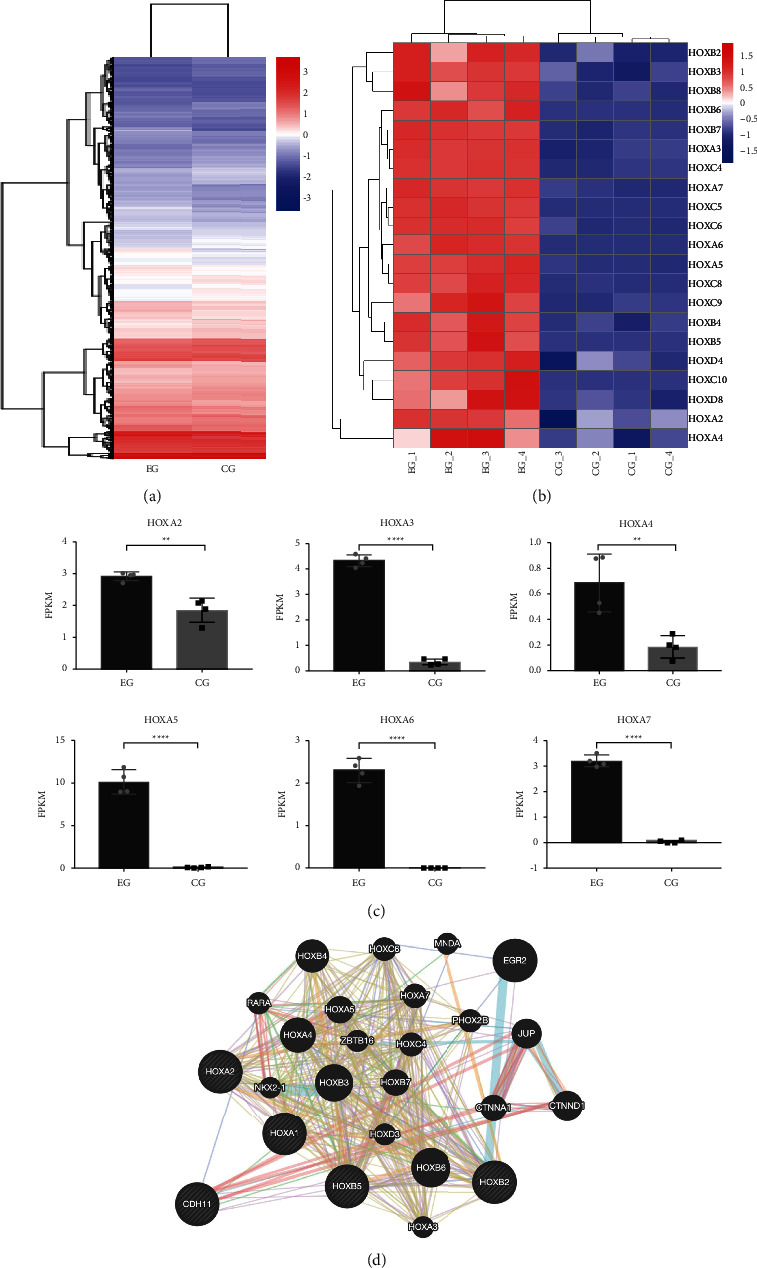
CDH11 is highly correlated with HOX family genes. (a) Cluster analysis of differentially expressed genes between the shCDH11 (EG) and shScramble (CG) groups. (b) Heat map of the microarray between EG and CG. (c) The expression of HOX family genes was measured by FPKM after CDH11 knockdown. (d) The diagram of the connections between CDH11 and HOX family members by GENEMANIA analysis. All experiments were performed at least three times. ^*∗∗*^*p* < 0.01; ^*∗∗∗∗*^*p* < 0.0001.

Furthermore, for a more comprehensive and in-depth understanding of CDH11 in microtia, we performed RNA-Seq on CDH11 knockdown chondrocytes (EG, *n* = 4) and shScramble controls (CG, *n* = 4) (Figure 7(a)). The differentially expressed genes data showed that HOX family genes were significantly upregulated in EG compared with CG (Figure 7(b)). In addition, the FPKM results showed that HOXA2, HOXA3, HOXA4, HOXA5, HOXA6, and HOXA7 were significantly increased after CDH11 knockdown (Figure 7(c)). Meanwhile, through GENEMANIA data, we found that CDH11 was also closely related to HOX family members (Figure 7(d)), suggesting a close association between CDH11 and HOX genes.

## 4. Discussion

Microtia is a congenital ear anomaly characterized by lacking all or part of the anatomical structure of the external ear, which is common in plastic surgery, but its etiology is poorly understood [15, 16]. Cadherins are a major class of membrane proteins with prominent roles in chondrocyte adhesion and the regulation of tissue organization and morphogenesis [17, 18]. In the present study, we found a significantly lower expression of CDH11 in microtia cartilage than in normal ear cartilage for the first time.

CDH11 is a type II cadherin, which enhances the firm adhesion between cells and ECM. Several studies have reported that CDH11 plays important roles in cell adhesion, growth, development, differentiation, recognition, and regulation of tissue and organ morphogenesis [19, 20]. Our previous studies demonstrated that CDH11 improved the mechanical strength of tissue-engineered elastic cartilage by promoting ECM synthesis and elastic fiber assembly. To verify the effect of CDH11 on chondrocyte biological function, both gain- and loss -of-function experiments were performed in vitro. The results showed that CDH11 promoted chondrocyte proliferation by increasing the S-phase chondrocyte numbers. CDH11 binds to and promotes chondrocyte proliferation both in vitro and in vivo, in line with our findings [21]. Migration and invasive properties are key biological characteristics that affect tissue and organ morphogenesis [22]. During ear development, the cranial neural crest chondrocytes undergo long-distance migration and eventually form the exact morphology of the auricle [23]. It has been confirmed that the malformation of the mandible and auricles caused by the TCOF1 gene mutation is closely related to the decreased number of migrating cells in the neural crest [24]. Therefore, migration capacity is a critical factor that affects ear formation. The wound healing and transwell experiments showed that CDH11 promoted chondrocyte migration, which may be an important aspect of how CDH11 participates in microtia occurrence. In our previous study, we demonstrated that the migration ability of microtia chondrocytes was reduced [25]. There was no significant difference in ECM synthesis between the microtia chondrocytes and healthy ones. So the migration ability maybe the predominant factor in the development of microtia associated with down-regulated CDH11.

Accumulating evidence has shown that, as an intercellular connecting molecule, CDH11 has been proven to be necessary for cellular organization, compaction, and matrix elaboration in some tissues [26]. It was proven by our in vivo experiments that CDH11 knockdown in chondrocytes worsened the tissue-engineered cartilage quality. As measured by histological examination, CDH11 knockdown decreased the expression of COL2A and ELN, which are the main components of ECM in cartilage. At the same time, the expression of SOX9 related to chondrogenic formation was also reduced when CDH11 was knocked down. These results suggested that CDH11 was involved in chondrogenesis and cartilage ECM synthesis. This is consistent with our previous finding that CDH11 improved the mechanical strength of tissue-engineered elastic cartilage by promoting ECM synthesis and elastic fiber assembly [27]. CDH11 has been reported to regulate collagen and elastin synthesis, affecting both the mechanical properties and contractile function of animal tissues such as the aorta, bladder, and skin [28]. Additional studies demonstrated that CDH11 KO mice produced less collagen and developed decreased colitis-driven intestinal fibrosis compared to controls [29]. The effect of CDH11 on chondrogenesis and matrix formation may be another important aspect of CDH11 in the occurrence of microtia.

Transcriptome sequencing analysis after CDH11 knockdown showed that the HOX family genes occupied the top most DEGs, and with the decrease in CDH11, the HOX family gene expression increased, which provided clues for the follow-up study of the role of CDH11 in the occurrence of microtia. HOX genes are important regulatory genes patterning head formation, including the development of the ear [30, 31]. For example, HOXA2 is a key transcription factor during the development of the second branchial arch, which makes a major contribution to the development of the external ear [32]. Si et al. [33] showed that HOXA2 could bind to a long-range enhancer and regulate expression of the HMX1 gene, which is a crucial transcription factor in eye and ear development, and reported the first two bilateral nonsyndromic microtia cases with HOXA2 mutations of Chinese origin [33]. Alasti et al. [34] ascertained that a mutation in HOXA2 is responsible for autosomal recessive microtia in an Iranian family [34]. Therefore, the present results indicated that CDH11 might be involved in the progression of microtia by regulating the expression levels of HOX family genes.

Taken together, the present study first verified the low expression of CDH11 in microtia cartilage and demonstrated an important role of CDH11 in proliferation, migration, chondrogenesis, and ECM synthesis of chondrocytes, which are vital chondrocyte properties for ear morphogenesis. Based on this understanding, CDH11 has the potential to enhance the biological activity of chondrocytes, supporting the hypothesis that CDH11 functions as an important gene in microtia.

## Figures and Tables

**Table 1:** Primer sequences for real-time PCR.

Primer name	Primer sequence (5′-3′)		Product size (bp)	
CDH11	Forward	GAGAATACAGGAGGCAGATA	149	
	Reverse	GTAGGTAGTCATAGTCCAAGTC		

COL2A	Forward	GGAAGAGCGGAGACTACTGGA	298	
	Reverse	TGGTAGGTGATGTTCTGGGAG		

ELN	Forward	GATCTTGGCGGAGCTGGTAT	342	
	Reverse	GAAGGGCTTGGGAGGTTTGC		

SOX9	Forward	CGGAGAAAGTCGGTGAAGAAC	176	
	Reverse	GTTGGTGGACCCTGGGATT		

GAPDH	Forward	GGCTACACTGAGGACCAGGTTG	117	
	Reverse	CCAGGAAATGAGCTTGACGAA		

Note. CDH11: cadherin-11; COL2A: collagen type II; ELN: elastin; SOX9: sry related hmg box-9.

## Data Availability

The data supporting the findings of this study are available from the corresponding author upon reasonable request.
